# Biochemical Properties and Neuroprotective Effects of Compounds in Various Species of Berries

**DOI:** 10.3390/molecules23010026

**Published:** 2017-12-22

**Authors:** Erin Kelly, Poorva Vyas, John T. Weber

**Affiliations:** School of Pharmacy, Memorial University of Newfoundland, St. John’s, NL A1B3V6, Canada; eekelly@mun.ca (E.K.); pv5843@mun.ca (P.V.)

**Keywords:** antioxidants, brain, neurodegeneration, neuroprotection, oxidative stress, polyphenols

## Abstract

Several species of berries, such as blueberries (*Vaccinium angustifolium*) and lingonberries (*Vaccinium vitis-idaea* L.), have attracted much scientific attention in recent years, especially due to their reported antioxidant and anti-inflammatory properties. Berries, as with other types of plants, have developed metabolic mechanisms to survive various environmental stresses, some of which involve reactive oxygen species. In addition, the fruits and leaves of berries have high amounts of polyphenols, such as flavonoids, which act as potent antioxidants. These compounds could potentially be beneficial for brain aging and neurodegenerative disorders. There are now several studies documenting the beneficial effects of various berries in cell models of neurotoxicity as well as in vivo models of neurodegenerative disease. In the current review, we discuss the metabolic strategies that plants and animals have developed in order to combat reactive oxygen species. We then discuss issues of bioavailability of various compounds in mammals and provide a synopsis of studies demonstrating the neuroprotective ability of berries and polyphenols. We also summarize findings from our own research group. For example, we have detected various polyphenols in samples of blueberries and lingonberries and have found that the leaves have a much higher antioxidant capacity than the fruits. Extracts from these species have also demonstrated neuroprotective effects in cellular models of toxicity and inflammation, which are being further pursued in animal models.

## 1. Introduction

Reactive oxygen species (ROS) are produced during normal metabolism in plants and animals. When the production of ROS is excessive this can lead to oxidative stress, which can be damaging to cells and organisms as a whole. Organisms have developed natural defense mechanisms to combat oxidative stress, but at times these mechanisms can become overwhelmed. Therefore in animals, it is beneficial to increase the amount of antioxidants in the body from external sources. Polyphenols are a group of compounds with high antioxidant capacity and are prevalent in the plant kingdom. Various species of berries contain high amounts of polyphenols, and therefore dietary intake of berries may increase antioxidants in the body and potentially protect against inflammation, cardiovascular disease and neurodegeneration. In this review, we discuss oxidative stress and mechanisms of combating this stress. We also provide an overview regarding the bioavailability of polyphenols found in berries, and the potential neuroprotective effects of these compounds.

## 2. Oxidative Stress and Reactive Oxygen Species

Although oxygen is a primary requirement of all forms of aerobic life on earth, its intermediates, when present in higher than normal concentrations in biological systems, may cause potential damage to cells that could ultimately lead to cell death. Any molecular species which contains one or more unpaired electrons in its outermost shell and can exist independently is called a free radical [1]. ROS may or may not be free radicals, and are intermediates of dioxygen formed when dioxygen is activated either by physical or chemical means [2]. When ground state triplet oxygen is physically activated by transfer of energy, the oxygen molecule gains enough energy to change its spin and become a ROS, i.e., singlet oxygen. Chemical activation occurs when an oxygen molecule is reduced step by step and the activated intermediate products are superoxide radicals, which is the first intermediate, and hydrogen peroxide, which itself is not very reactive but it is reduced to the highly reactive hydroxyl radical [3]. Production of ROS during respiration is unavoidable since the mitochondrial electron transport chain involves direct reduction of oxygen by the free energy of electrons [4]. Out of the total O_2_ consumption by plant tissue, an estimated 1% contributes to ROS generation [5]. In mitochondria, complex I and III are known to be responsible for ROS production, particularly the superoxide anion which is dismutated to a less toxic but more diffusible form of ROS, i.e., hydrogen peroxide [6]. Hydrogen peroxide penetrates through the mitochondrial membrane and can also give rise to the more toxic hydroxyl radical [7]. Hydroxyl radicals formed in the mitochondria can damage mitochondrial proteins, lipids and DNA [8].

There are several external factors that can enhance oxidative stress and hence increase the formation of ROS under stress conditions in plants. These factors include atmospheric pollutants such as ozone and sulphur dioxide, accumulation of heavy metals, herbicides, as well as draught conditions. ROS are also formed in other organisms including human beings as a part of normal metabolic processes and as a result of several external factors such as smoke, pollution, exposure to industrial chemicals, ozone, ultra violet radiation, X-ray, pesticides and certain drugs [9,10]. Some internal sources of ROS generation in human beings include the mitochondria, peroxisomes, inflammation, xanthine oxidase and exercise [10]. ROS in biological systems contribute to damaging cellular proteins, DNA and lipids [11].

## 3. Defense Mechanisms against Oxidative Damage

Plants are immobile and cannot escape from adverse environmental effects as easily as other organisms such as animals. For this reason, plant systems have adapted to survive in extreme conditions, e.g., illumination, high temperature, draught, and freezing, which could lead to oxidative damage due to excessive production of free radicals. These developed antioxidative mechanisms involve a wide array of compounds, which includes superoxide dismutase (SOD), catalase, and important antioxidant metabolites such as ascorbate, glutathione, tocopherol, carotinoids and phenolic compounds such as flavonoids and anthocyanins.

SOD is a class of metalloenzymes which catalyzes the dismutation of superoxide to hydrogen peroxide in aerobic and anaerobic organisms [12], and is considered to be the first line of defense against ROS since the superoxide radical is the first reduction product of oxygen [13]. Catalytic dismutation of superoxide by SOD occurs at a rate 10,000 times faster than spontaneous dismutation [6]. SODs are distributed in almost all cellular compartments in plant tissue including the cytosol, mitochondria, chloroplasts, peroxisomes and the extracellular space [14]. Hydrogen peroxide, produced by the action of SOD, is a ROS as well and quenching of this diffusible reactive species is important to maintain its low level in the cell to prevent damage. Catalase is one of the most important enzymes devoted to regulating levels of hydrogen peroxide in cells [15]. The ascorbate–glutathione cycle is essential for maintaining a proper redox level in plants and other organisms. Ascorbate or ascorbic acid, also known as vitamin C, is one of the most important antioxidants in biological systems [16], and it can be synthesized by all plants and animals, except primates and guinea pigs [17]. Plants contain ascorbic acid in all cell types where it can accumulate in millimolar quantities [14]. Ascorbate detoxifies ROS by directly reacting with hydroxyl radicals, superoxide, and singlet oxygen [18], and is very efficient in inhibiting peroxidation in human plasma lipids, more than any other plasma components such as alpha tocopherol [19]. Glutathione is a tripeptide and is an abundant compound in plants found in almost all cell compartments [20]. It serves as a reducing substrate in the ascorbate-glutathione pathway and is a very important antioxidant, which helps maintains redox levels in both plants and animals [21]. Carotenoids are a class of natural pigments which are widespread in many fruits and vegetables and are lipid soluble molecules, which serve as important antioxidants in both plants and animals as well [2]. Tocopherol is a lipid soluble compound mainly localized in plastids. It is a very important dietary source of nutrients for humans and other animals since it is only synthesized in plants [22]. Tocopherol is a crucial antioxidant in plant systems since it is capable of terminating the free radical chain, which causes lipid peroxidation in food and biological systems by reacting with organic peroxyl radicals. Hence tocopherol functions as an antioxidant in vivo by protecting poly unsaturated fatty acids (PUFA) from reactive oxygen damage [23].

## 4. Flavonoids

Flavonoids are a large class of low molecular weight secondary metabolites found ubiquitously in higher plants and are present in almost all plant compartments from the roots, to flowers and fruits [24]. Red fruits, citrus fruits, apple, onion, chocolate, red wine, and tea are rich dietary sources of flavonoids [25,26,27,28,29]. Flavonoids are divided into 14 different groups [30], which include the flavones, isoflavones, flavanones, flavonols, flavanols (catechins), and anthocyanidincs. Flavonoids have been reported to have widespread biological functions including plant pathogen interactions, pollination and seed development [31] but arguably, the most important property of flavonoids in biological systems is their antioxidant abilities [32]. Flavonoids have properties of inhibiting auto-oxidation and scavenging free radicals [33] and can act as antioxidants pertaining to their ability to transfer electrons to free radicals and chelate metal catalysts [34]. During biotic and abiotic stress conditions such as drought, wounding and metal toxicity, many flavonoid biosynthetic genes are induced and flavonoid levels increase [35,36].

Anthocyanins belong to the flavonoid class, have strong hydrophilic properties, and are responsible for red and blue colors in fruits, vegetable and flowers [37]. In addition to being natural pigments, anthocyanins are potent antioxidants [38] and have the ability to prevent lipid oxidation [39] and scavenge free radicals [40]. Dietary intake of fruit and vegetables has been reported to have beneficial effects on human health such as anti-cancer and anti-aging properties [41,42,43,44,45]. Anthocyanins are also important for improving the nutritional values of processed foods [46,47].

## 5. The Plant Family Ericaceae as a Source of Antioxidants

The natural defense mechanisms that combat oxidative stress in plants are very similar to the mechanisms seen in animals, including humans. These include high endogenous levels of SOD, catalase and glutathione, for example [11]. At times, these natural defense mechanisms may be saturated and therefore it is necessary to increase levels of antioxidants through dietary means. Ericaceae is a plant family that is a very rich source of antioxidants. This family is highly ornamental and includes mostly shrubs and small trees. In Canada, 18 genera represent some of the most important native medicinal and edible plants, which include blueberry, bilberry, cranberry, labrador tea and bearberry [48]. It has been reported that the Ericaceae family is amongst the top 10 families of plants used in traditional medicine [49] and that the use of Ericaceae as medicinal plants is significantly higher than expected based on the number of species present in the plant kingdom [50].

The *Vaccinium* genus of Ericaceae comprises 400 species including *Vaccinium angustifolium* (Lowbush Blueberry), *Vaccinium myrtilloides* (Canadian Blueberry), *Vaccinium myrtillus* L. (Bilberry), *Vaccinium oxycoccos* (cranberry), *Vaccinium parvifolium* (Red Huckleberry) and *Vaccinium vitis-idaea* L. (Cowberry, Lingonberry, Partridgeberry). Blueberries, cranberries and lingonberries are also commercially cultivated and are quite economically important. *Vaccinium* species are characterized by fleshy fruits with high ascorbate and anthocyanins levels [51,52].

## 6. Bioavailability Issues

The ingestion of plants rich in polyphenolic compounds has been documented to have positive effects on various systems in the body, including the gastrointestinal tract and the cardiovascular system [52]. Several studies have been conducted to analyze the bioavailability of such compounds. For example, several anthocyanin metabolites have been detected in the urine of individuals who ingested blueberry juice [53,54], and therefore were in high concentration in the gastrointestinal tract. The anthocyanin cyanidin-3-glucoside has also been detected in human urine after ingestion [55] and cyanidin-3-*O*-galactoside and related metabolites have been found in urine after ingestion of lingonberries [56]. Other studies have detected anthocyanins in serum, for example Kay et al. detected several metabolites in the serum of volunteers after ingesting an extract from chokeberry [57]. Cyanidin-3-glucoside and cyanidin-3,5-diglucoside have been detected in the serum and liver of humans and rats after dietary supplementation of these compounds [58].

There is now substantial evidence suggesting that the ingestion of diets high in berries can have positive effects on the brain [59,60,61], it is still controversial whether this is due to direct or indirect effects on nervous system tissue [62]. Although most polyphenols that have been analyzed can be absorbed from the gastrointestinal tract and then distributed to blood and tissues, they additionally must be able to get across the blood-brain barrier in order to have a direct effect on the brain. Some research has demonstrated that dietary polyphenols can cross the blood-brain barrier [59], and anthocyanins specifically have been detected in brain tissue after oral administration to rodents [63,64,65] and pigs [66,67]. From these aforementioned studies, estimates of specific anthocyanins in brain tissue are in the sub-nanomolar range (~0.2–0.25 nmol/g tissue) [64,65], or even as low as the femtomolar range [67]. It is also possible that polyphenolic compounds contained in extracts that are tested in vitro may not be the predominate forms that would actually enter the brain. Although anthocyanins arguably have good bioavailability, they do undergo significant metabolism, producing diverse metabolites [53,55,62]. Some evidence suggests that certain polyphenolic compounds are maintained in their natural glycosylated form [64,65]. Xenobiotic metabolism also likely contributes to the amounts and forms of polyphenols that cross the blood-brain barrier, as additional evidence has demonstrated that glucuronide forms of anthocyanins can be detected in the brain [67]. It is also possible that metabolites of anthocyanins may compete for entry into the brain [62] and therefore more well-designed studies on the ability of anthocyanins to cross the blood-brain barrier are necessary. These issues of bioavailability in the nervous system should be taken into consideration for in vivo and in vitro studies involving polyphenols in berries.

## 7. Neurodegenerative Mechanisms Associated with ROS and Effects of Berry Products In Vivo

Currently, there is no cure for neurodegenerative diseases such as Parkinson’s disease (PD), Alzheimer’s disease (AD), and Huntington’s disease (HD) [68]. There are drugs and therapies available but they only help relieve the symptoms of the disease and have no direct effect on its pathology [69,70]. Only a small portion of cases are caused by environmental or genetic factors, most cases are sporadic or idiopathic and are very hard to diagnose [69,71]. Identifying a potential drug or compound which is able to not only prevent neuronal loss, but reverse some of the damage is extremely difficult but very important. Nerve degeneration is also common even when a neurodegenerative disease is not present. Over time, the body loses its ability to maintain and produce new nervous tissue as we age, and this can lead to many cognitive and motor impairments. We are now in an era where the world’s population is living longer than ever, so age-related diseases are becoming more and more common [68,72].

As previously described, polyphenols are botanical nutraceuticals which have antioxidant properties. This characteristic is what makes polyphenols important to neuroprotective studies. Along with their antioxidant properties against ROS, polyphenols are known to have other health benefits such as anti-inflammatory, anti-cancer, anti-ulcer, and anti-infective properties [52,73,74]. Polyphenols may also have indirect peripheral effects [72], such as improving cardiovascular health resulting in increased blood flow to the brain [75,76]. Some antioxidants are found in the body naturally while others need to be obtained in the diet [73].

Berry species (e.g., the *Vaccinium* genus) are arguably the most researched and have been proven to contain some of the highest levels of polyphenols compared to other plant species [77]. Blueberries, in particular, have been shown in several studies to have high levels of polyphenols and to be the most neuroprotective [73,78]. The use of botanical nutraceuticals for their antioxidant properties and overall health benefits have been investigated not only within the *Vaccinium* genus but also all across the plant kingdom [73]. In addition, several berries not in the *Vaccinium* genus have high antioxidant capacity, such as crowberries (*Empetrum nigrum* L., [79]). Polyphenols can be classified into five major groups: diferuloylmethanes, stilbenes, flavonoids, phenolic acids and tannins [80]. From a chemical point of view, flavonoids are the main compounds responsible for the antioxidant activity of berries [80], more specifically the anthocyanins and flavonols [81]. Anthocyanins have been identified in several brain areas and have been associated with neuroregeneration and protection [64,82]. Berries are one of the best options for polyphenol related neuroprotective studies because they contain several different kinds of these compounds that can work together and have a synergistic effect in the central nervous system (CNS) [83]. This is important, especially with regard to localization of a particular antioxidant in the brain [78], and for activating neuroprotective response pathways [70]. Berries are also very promising because they have more than one mechanism of action when it comes to neuroprotection [70]. Antioxidants are not taken up equally into all brain areas either. For example, uptake of vitamin E, a potent antioxidant not found in berries, is lower in the striatum compared to other brain areas [78].

Antioxidants are essential to scavenge free radicals and not only protect the CNS from further degradation but potentially reverse some of the damage that has already been done. It has been suggested that one of the main mechanisms of neurodegeneration is from the increased and long-term effects of oxidative stress. As described earlier, oxidative stress in the CNS is caused when harmful ROS build up faster than the endogenous redox reactions can eliminate them [69]. Disruption of the sensitive regulation of ROS and balance of antioxidants can lead to cell death and damage [68]. A similar process called nitrosative stress can also play a role. Nitrosative stress is when reactive nitrogen species (RNS) build up in the cell and additively contribute to the free radical load. ROS and RNS are missing one or more electrons so they attack other molecules, such as cell structures, membranes, and DNA to replace their missing charge. This results in extensive cell damage and eventual cell death. Antioxidants are electron donors and are able to neutralize these ROS/RNS without becoming free radicals themselves [11,75]. The efficacy of antioxidants comes from their chemical structure, composed of one or more aromatic rings with several hydroxyl groups attached. These hydroxyl groups trap excess electrons balancing ROS/RNS [80]. Certain antioxidants based on their chemical structure, i.e., the number of rings and the chemical element that binds them together, may be better at trapping excess electrons and be a stronger antioxidant. The structure also plays a role when getting through the blood-brain barrier, which depends on a number of factors such as size and lipophilicity [62,83,84]. The brain is particularly susceptible to oxidative stress because of its high demand for oxygen [69]. Due to neurons’ high cellular respiration rate, and low antioxidant defense system they are also highly vulnerable to oxidative stress particularly in an aging brain [68].

Oxidative stress can have an effect on lipids in the membrane of mammalian cells as well. Free radicals can build up making the membrane faulty. This results in the cell spilling its contents into the surrounding area causing further inflammation and promoting the damage of more cells. Free radical builds up in the membrane can also promote permeability to Ca^2+^ [69]. This can cause changes in normal cell function by over-activating Ca^2+^-dependent enzymes, such as phospholipase A2, a precursor to arachidonic acid which is responsible for mediating inflammatory responses [85]. Excess Ca^2+^ also promotes the production of free radicals in the cell and other harmful compounds such as peroxynitrate and hydrogen peroxide [86]. Elevated levels of Ca^2+^ can activate endonucleases that degrade DNA and protein, as well as cause damage to the mitochondria, which again results in the production of free radicals [85,86]. Also, when the mitochondria is damaged this allows for an influx of Ca^2+^ ions through the mitochondrial membrane. This activates caspases, which are enzymes responsible for triggering apoptosis. This was shown in a study where polyphenols, specifically mangiferin and morin were administered. The result was a reduction of apoptosis by limiting the activation of the glutamate receptor responsible for activating caspase-3 neurons in vitro and in vivo [74,87]. Neurodegeneration in the brain can also be induced by glutamate-mediated excitotoxicity. Glutamate is an essential excitatory neurotransmitter in the CNS essential for sending chemical messages. Glutamate-mediated excitotoxicity causes nerve damage and death by the excessive stimulation of glutamate receptors which leads to oxidative stress [72,73].

In vivo studies mainly use rodents for investigation. The animals are usually supplemented with a berry-enriched diet and then evaluated on their cognitive and motor functions. These studies are more difficult to conduct as they are typically more expensive and have higher variability than in vitro studies [75]. Blackberries (*Rubus* subgenus *Rubus*) have been tested in vivo, where rats were given a 2% blackberry-enriched diet for 8 weeks. This resulted in an overall improvement of motor coordination and memory, as well as some neuronal damage reversal via the increase of receptor sensitivity and free radical scavenging [88]. Another study from this research group demonstrated that a blueberry-enriched diet could reverse age-related memory loss in rats [89]. Bilberries have also been used in neurodegenerative research. More specifically, they are used in diabetes-related research to try to prevent and repair some of the brain damage caused by diabetes, particularly tissue loss and reduction in cognitive function and memory [90]. It was found that bilberries were able to modulate neurodegeneration in diabetic rats by increasing neurotransmitter release and reducing the quantity of ROS in the brain [90]. The researchers also note that the extract was able to promote healthy morphological modulations in αCaMKII in hippocampal neurons.

Casadesus et al. analyzed the effects of polyphenols in regard to hippocampal plasticity and cognitive behavior, specifically age-related CNS deterioration associated with the loss of learning and memory [91]. They found that a short-term blueberry diet administered to aging rats improved hippocampal neuroplasticity and had a positive effect on spatial memory by promoting the activation of insulin-like growth factor 1 (IGF1) and IGF-1 receptor levels, which mediate the rate of neurogenesis. In a study conducted by Joseph et al. rats were fed a diet containing blueberry, spinach or strawberry extract for eight weeks [78]. The rats given a polyphenol-enriched diet exhibited a better performance for the rotarod and Morris water maze trials, concluding that the diet was able to increase cognitive and motor function. They also showed that the blueberry supplementation was the only diet capable of significantly reversing some of the neurodegenerative effects related to aging. A clinical study conducted in 2010 investigated the effects of daily wild blueberry juice consumption for 12 weeks in adults experiencing early memory decline [82]. Regarding list recall and paired associate learning, the group administered the berry juice performed exceptionally better than the placebo group. Even though these preliminary results are encouraging, the study is highly debatable however as the sample size was only nine [82]. A recent study showed more convincingly that a diet high in blueberries can improve cognitive abilities in older adults [61]. However, further trials and clinical experiments need to be conducted.

## 8. Examination of Effects of Berry Products on Disease-Related ROS Mechanisms In Vitro

The majority of studies investigating the neuroprotective role of polyphenols are conducted in vitro. This is mainly because these models more accessible, fairly inexpensive and provide more rapid results than in vivo models [92]. It is also easier to create appropriate models of neurodegenerative disease and use specific cell lines of interest. Studies have been conducted to investigate neuroprotective potential in a number of various plant species, including grape seeds [70], walnuts [93], plums [70], and myrtle [94]. These studies have indeed been positive, most likely due to antioxidant capacity of compounds in these foods. Research has also been conducted with different species of berries. For example, a study done by Casedas et al. concluded that both cranberry (*Vaccinium macrocarpon*) and blueberry (*Vaccinium myrtillus*) juices are beneficial for diseases where oxidative stress is prominent [95]. They found that both extracts were very high in anthocyanins, blueberries being slightly higher with no statistical significance. Blueberries also contained a higher variety of anthocyanins compared to the cranberries. Both were neuroprotective against hydrogen peroxide toxicity and were able to scavenge 2,2-diphenyl-1-picrylhydrazyl (DPPH) and superoxide radicals [95]. Tavares et al. investigated the effects of digested polyphenols from two species of wild blackberries (*Rubus brigantinus* and *Rubus vagabundus*) in a neurodegenerative cell model [81]. The blackberry metabolites were effectively able to reduce levels of ROS and activate cellular stress pathways in the CNS [83]. Mulberry (*Broussonetia papyrifera*) extracts were also found to reduce damage caused by rotenone in vitro [70].

AD and PD can be recognized by the aggregation of amyloid-beta peptide (Aβ) which is a result of oxidative stress in the CNS [77], and studies of this protein in vitro are steadily increasing. The Aβ peptide can be toxic to neurons in high levels and induce apoptosis, mitochondria dysfunction and activation of the nuclear factor, NF-κB, which is a group of proteins largely involved in inflammatory responses [69]. When the Aβ protein acts irregularly and begins to aggregate, these masses are responsible for slowing and blocking neuronal communication particularly involved with memory and cognition [77]. These aggregates are also capable of turning metals into ROS, however, polyphenols are capable of ion chelation which helps detoxify the CNS [69]. Fuentealba et al. analyzed the neuroprotective potential of blueberry extract in a neurodegenerative in vitro model of Aβ [96]. They noted that the blueberry extract was capable of preventing mitochondrial damage associated with Aβ. As well as reduce the quantity of Aβ present in the brain by inhibiting its aggregation and reducing neuroinflammation through regulation of NF-κB. Similar results were also observed by Papandreou et al. where blueberry polyphenols were able to reduce Aβ aggregation by ~70% in vitro [97]. In other studies, polyphenols from partridgeberries (lingonberries) protected neurons in vitro from Aβ toxicity [96], and blueberry leaf extracts also protected cells from Aβ toxicity [98].

Alpha-synuclein (α-synuclein) is a presynaptic protein abundant in the brain, however, its function is poorly understood [70]. When electrons in the mitochondrial electron transport chain are disrupted by neurodegeneration this promotes the abnormal aggregation of α-synuclein. These aggregates, referred to as Lewy Bodies, are associated with neuronal damage and death. Alpha-synuclein can also contribute to neurodegeneration when Lewy Bodies are released from dead or dying neurons, where they can activate surrounding microglia and promote the release of inflammatory mediators [99]. Microglia are the innate immune cells in the CNS which usually remain in a resting state but can be activated by different cues such as brain trauma or environmental stimuli [99]. When microglia become activated they transform morphologically and are responsible for regulating brain function and cell survival [99]. However, microglia can become over-activated which induces the overproduction of compounds that in high levels can become toxic and cause extensive damage. For example, inflammatory proteins such as tumor necrosis factor-α (TNF-α), which is also a marker of microglia activation [72], can be upregulated and released into the surrounding area causing other microglia to become over-activated as well, further contributing to neurodegeneration. Polyphenols are able to target and inhibit microglial signaling pathways before they become activated and release excess inflammatory mediators [72]. The proposed mechanism is that polyphenols are able to increase levels of IκBα, a protein that inhibits NF-κB translocating from the cytoplasm to the nucleus, therefore preventing the transcription of DNA associated with inflammatory mediators [72]. Polyphenols such as resveratrol (e.g., found in grapes, peanuts and some species of berries), along with scavenging of free radicals, potentially stimulate genes that are responsible for the activation of endogenous antioxidant pathways as well [86]. When looking at the brains of deceased patients diagnosed with PD, a reduction in the activity of nicotinamide adenine dinucleotide (NADH) dehydrogenase can be observed [70]. NADH dehydrogenase is an enzyme in the mitochondria responsible for the transfer of electrons. Because PD affects this site in the mitochondrial electron transport chain it allows electrons to escape, leading to an increase in ROS [70]. Interference with the mitochondria, which is responsible for generating energy, reduces the overall productivity of the cell as well [74]. It has been observed that berry extracts with high levels of anthocyanin and proanthocyanin were able to provide protection against mitochondrial damage and reduce microglial activation in response to the pesticide rotenone, a cell culture model of PD [70]. The proposed mechanism of action is that the polyphenols are able to displace rotenone from its binding site on NADH dehydrogenase, resulting in the reduction of ROS produced [70].

As discussed earlier, polyphenols undergo extensive metabolism and it is important to study the potential protective effects of metabolites in addition to parent compounds (see Table 1). Polyphenol metabolites were investigated by Figueria et al. where glutamate excitotoxicity conditions were present in a 3D in vitro model containing neurons and astrocytes [72]. The metabolites exhibited neuroprotective effects in these cultures, and the study showed that the metabolites are able to cross the blood-brain barrier and interfere with microglia activation and inflammation. The majority of the in vitro studies do not take polyphenols extensive metabolism into account, or their specific pharmacokinetics and pharmacodynamics [80,100]. Therefore, by this study analyzing the metabolites of common polyphenols, it provides beneficial insight on the compounds that are actually reaching brain cells. It also provides a better understanding of polyphenol metabolism once inside the CNS, the quantity of these metabolites as well as their mechanism of action and particular role in neuroprotection [72].

In addition to issues associated with the metabolism of polyphenols, there are other factors that should be taken into consideration when using in vitro models for neuroprotective studies (see Table 1). For example, using concentrations of polyphenolic compounds that realistically can enter the brain and reach target cells is essential, but this has to be estimated based on the limited number of bioavailibity studies discussed earlier. Also, the use of cell lines can be convenient, but may not represent nerve cells as accurately as primary cells, which are dissected directly from rodent brains. This concept was evident in a study conducted by Papandreou et al., where it was found that different polyphenols reacted a certain way according to the characteristics of a specific cell line [77]. For example, some cell lines required a higher concentration of berry extract than others to elicit protection against hydrogen peroxide-mediated cell death. However, the advantages of in vitro models cannot be overlooked, such as analyzing several compounds much more rapidly than in vivo studies. For example, Strathearn et al. showed that extracts high in anthocyanins and proanthocyanins were able to better suppress the neurodegenerative effects of rotenone in a cell model of PD compared to other phenolic compounds [70]. Studies such as these can aid in leading to appropriate in vivo experiments.

## 9. Present and Future Laboratory Studies

In our own research group, we identified a total of 21 phenolic compounds when investigating blueberries (*Vaccinium angustifolium*), lingonberries (*Vaccinium vitis-idaea*) and black currants (*Ribes lacustre*) native to Newfoundland and Labrador [81]. Blueberries contained 11 of the identified compounds, while lingonberry and black currant contained six. In this study, both blueberries and lingonberries were confirmed to be highly protective against trauma in vitro, which suggests that they are potentially useful for further neuroprotective studies, particularly in vivo studies. We also studied the neuroprotective effects of blueberry and lingonberry extracts against glutamate-mediated excitotoxicity and determined that for this particular mechanism of neurodegeneration blueberry fruit extracts were extremely protective whereas lingonberry fruit extracts were less effective [73]. It is suggested that this may be due to the chemical profile of lingonberry fruit; specific phenolic compounds found in the blueberries may be better at protecting neurons and glia compared to those found in the lingonberry fruit. We also conducted research on the neuroprotective potential of leaves from *Vaccinium* species [73]. It was determined that in addition to the fruits containing high levels of phenolic compounds in both blueberries and lingonberries, the leaves exhibited much higher levels and proved to be even more protective in an in vitro model of glutamate-mediated excitotoxicity. Therefore, moving forward it would seem beneficial for studies to also include and investigate other plant components instead of just the fruits.

There is now ample evidence, in our view, of the benefit of berry-derived polyphenols for neurodegeneration and the brain as a whole. However, several of the latter studies discussed in vivo point out the importance of understanding bioavailability. For example, how much fruit would need to be consumed daily to provide beneficial effects against brain aging or neurodegenerative disease is not known. Also, it is not known how long the beneficial effects of a diet rich in berries lasts after the berries have been consumed. Further development of nutraceuticals containing extracts from fruits and leaves may be desirable. However, further investigation is needed to determine the appropriate ‘dose’ for health benefits or treatment of a particular disease, without resulting in toxicity. Studies in vitro need to use appropriate concentrations of polyphenols that are predicted to enter the brain and reach target cells. In our own laboratory group, we are further pursuing studies in vitro and in vivo so that we may have a greater understanding of the mechanisms by which polyphenols produce their beneficial effects on the nervous system.

## Figures and Tables

**Table 1:** Advantages and disadvantages to in vivo and in vitro experimental approaches to studying polyphenols in models of neurodegeneration.

Type of Model (s)	Advantages	Disadvantages
In vivo	Can potentially evaluate the effects of compounds or metabolites in specific brain areas.	Are often more expensive than in vitro studies due to animal maintenance and other costs, including use of transgenic and mutant models.
	Can determine effects of compounds using behavioral experiments or outcomes.	Can take much longer to screen potential neuroprotective effects of polyphenols than in vitro studies.
	Can measure the extent to which polyphenols in the diet and their metabolites enter the brain.	
In vitro	Are often less expensive than in vivo models.	Cytoarchitecture of the brain is not maintained.
	Can quickly screen polyphenols for neuroprotective potential.	Compounds may be tested at concentrations not achieved in nervous system tissue.
	Can more easily discern the cellular effects of compounds and their mechanisms of action.	Cell lines have been somehow genetically modified, which may alter experimental results.

This table was partially adapted from Weber, 2015 [92].
